# A Case of Autoimmune Polyendocrine Syndrome Type 3B and Peripheral Neuropathy Due to Thiamine Deficiency

**DOI:** 10.7759/cureus.52123

**Published:** 2024-01-11

**Authors:** Asuka Suzuki, Koji Hayashi, Maho Hayashi, Yuka Nakaya, Mamiko Sato

**Affiliations:** 1 Department of Rehabilitation Medicine, Fukui General Hospital, Fukui, JPN; 2 Department of Internal Medicine, Fukui General Hospital, Fukui, JPN

**Keywords:** thyroid gland, vitamin b12, polyneuropathy, thiamine, autoimmune polyendocrine syndromes

## Abstract

Autoimmune polyendocrine syndrome (APS) type 3B is characterized by presence of autoimmune thyroid disease, chronic atrophic gastritis and pernicious anemia. In this report, we present a rare case of APS type 3B with neuropathy by thiamine deficiency. A 65-year-old man had a history with hypothyroidism, gastritis, gastrectomy for gastric cancer and subacute combined degeneration of the spinal cord. Patient developed polyneuropathy with not mecobalamin but thiamine deficiency. Serum anti-thyroglobin (TG), anti-thyroid peroxidase (TPO), and anti-gastric parietal cell antibodies were positive. He was treated with thiamine supplementation and improved muscle weakness, sensory impairment and gait disturbance. Classically, it is reported gastric cancer related to hypothyroidism. Additionally, thiamine deficiency can be caused by gastrectomy. Here, his thiamine deficiency was related to APS type 3B, leading to polyneuropathy.

## Introduction

Autoimmune polyendocrine syndrome (APS) comprises a diverse group of clinical conditions. It is characterized by functional impairment of multiple endocrine glands due to loss of immune tolerance [1]. APS type 3 is characterized by presence of autoimmune thyroid disease (Hashimoto's thyroiditis, idiopathic myxedema, Graves' disease, asymptomatic thyroiditis, or endocrine exophthalmus). Type 1 diabetes mellitus (type 3A), chronic atrophic gastritis, pernicious anemia (type 3B), vitiligo, alopecia, and myasthenia gravis (type 3C) are also characteristics of APS type 3 [2,3]. Herein, we report a case of APS type 3B associated with polyneuropathies with thiamine deficiency after gastrectomy for cancer. 

## Case presentation

A 65-year-old Japanese man with a medical history of hypothyroidism at 52 years of age; intestinal obstruction and partial colon resection at 62 years of age; and chronic gastritis and gastrectomy for gastric cancer at 64 years of age experienced gait disorder and lower limb muscle weakness. The patient had no smoking history. The patient had a history of alcohol consumption (ethanol amount: 20-40 ml/day), but dietary intake was good. The patient consulted with neurologists and was diagnosed with macrocytic anemia and subacute combined degeneration of the spinal cord (SCDS) due to gait disturbance, disturbance of deep sensation, and decreased serum vitamin B12 levels (226 pg/mL, normal range: 233-914). The result of plane MRI in the thoracic and lumbar spine revealed no abnormalities in the spinal cord. He was administered oral and intravenous vitamin B12 (intravenous: 1000 mg/day, four weeks) and his gait disturbance was improved. After discharge from the hospital, vitamin B12 was supplemented intravenously at each outpatient visit. Blood vitamin B12 concentration remained within the normal range.

**Figure 1:**
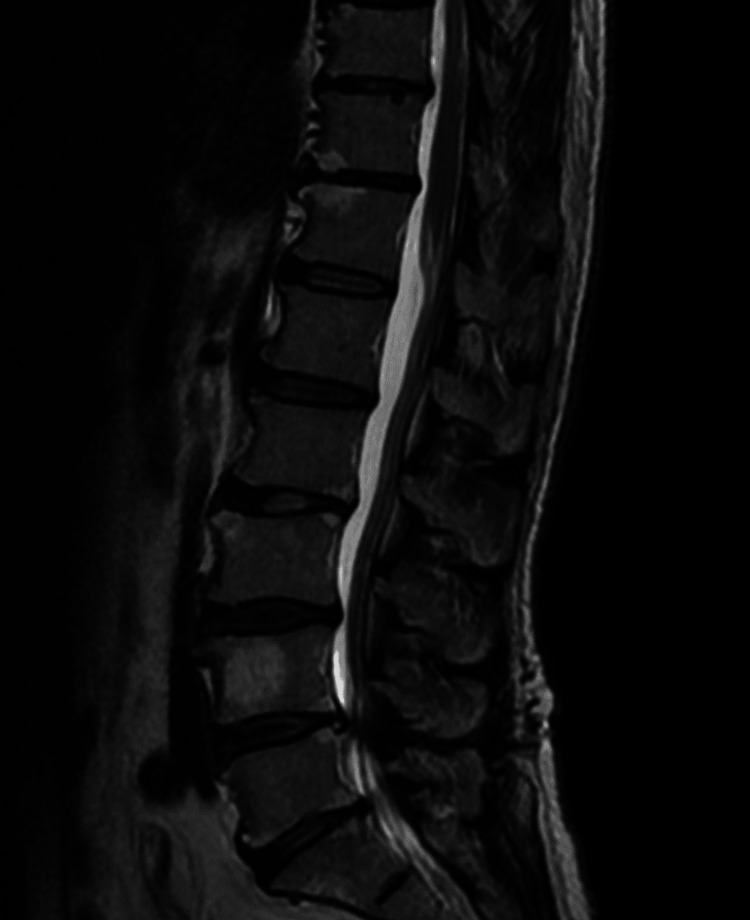
The result of lumbar magnetic resonance imaging (MRI). Lumbar MRI showing a herniation at the L4/5 level.

**Figure 2:**
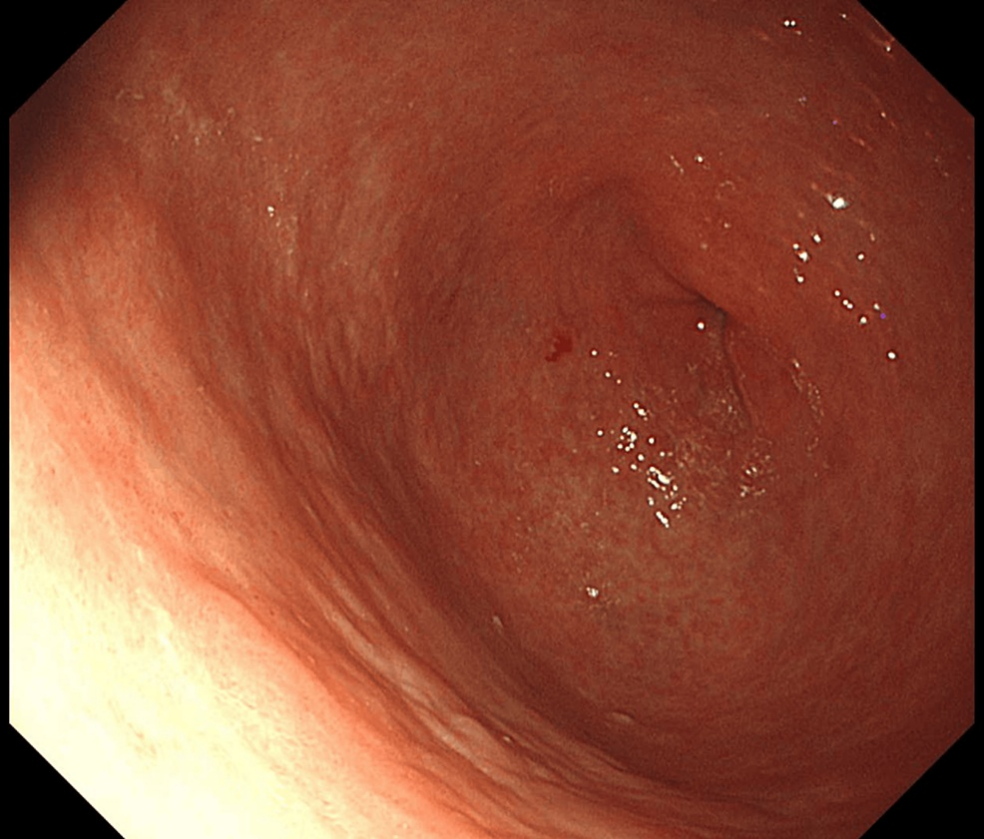
The result of endoscopic upper gastrointestinal endoscopy. Endoscopic upper gastrointestinal endoscopy showing atrophic gastritis in the residual stomach after gastrectomy.

**Figure 3:**
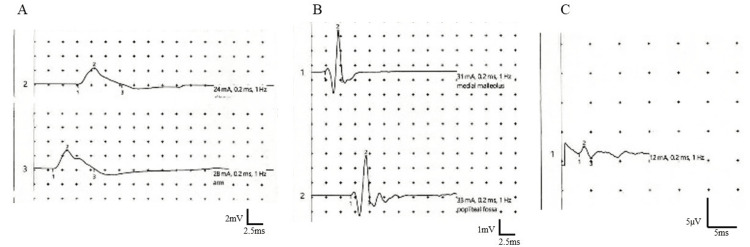
The results of nerve conduction studies. Motor nerve conduction study in the median nerve (A) and the tibial nerve (B) showing decreased compound muscle action potentials. Sensory nerve conduction study in the sural nerve (C) showing decreased a sensory nerve action potential.

At age 66 years, four weeks before admission, he developed a gait disorder and weakness in the lower limbs and visited our hospital. Although his serum vitamin B12 was normal range (429 pg/mL), oral and intravenous supplementation of vitamin B12 was retried for three weeks. Despite receiving vitamin B12 injections, gait disturbance and weakness in both lower limbs were not improved but exacerbated. One week before admission, the patient developed loss of appetite, making eating difficult. Since the gait disturbance and appetite loss became highly severe, the patient was admitted to our hospital for close examination and treatment. The patient’s vital signs were unremarkable. We did not identify thyroid gland enlargement or anemia checked in the palpebral conjunctiva. Neurological examination revealed lower-extremity-dominant and distal-dominant muscle weakness (MRC grade 4); areflexia in the lower extremities; decreased superficial sensation around the umbilicus; distal-dominant dysesthesia in the lower limbs; decreased position sense in the toes and vibratory perception in the medial malleolus; and orthostatic hypotension. Blood test results were unremarkable except for mild normocytic anemia (hemoglobin, 12.6 g/dL); positivity for anti-thyroglobin (TG), anti-thyroid peroxidase (TPO), and anti-gastric parietal cell antibodies; and decreased vitamin B1 (14.5 ng/mL, normal range: 21.3-81.9 ng/dL). Vitamin B12 levels increased to 1039 pg/mL (normal, 233-914 pg/mL). Thyroid markers, including free T4 and thyroid stimulating hormone, revealed euthyroid state. Serum intrinsic factor antibodies were negative. Cerebrospinal fluid analysis showed normal results. Lumbar magnetic resonance imaging showed lumbar disc herniation at the L4/5 level (Figure 1). An upper gastrointestinal endoscopy revealed atrophic gastritis (Figure 2). Gastric biopsies were performed to rule out amyloidosis. However, amyloid deposition was not observed. Additionally, a retrospective pathological analysis of a gastric resection specimen at 64 years of age was non-diagnostic for amyloidosis. Although nerve conduction studies revealed no conduction block or temporal dispersion, decreased compound muscle action potentials and sensory nerve action potentials were found (Figure 3). The patient was treated with intravenous vitamin B12 at first, followed by intravenous vitamin B1 after diagnosis of thiamine deficiency. On day 22, serum vitamin B1 levels increased to 193.9 ng/mL. Muscle weakness and sensory impairment including superficial and deep sensation improved after vitamin B1 supplementation, and gait disturbance gradually improved with combined rehabilitation. Finally, the patient was able to walk without assistance on day 60. On day 70, the patient was discharged from our hospital.

## Discussion

This report describes a rare case of APS type 3B that developed neuropathies. The patient had a history of SCDS due to vitamin B12 deficiency at age 65 years. The neurological symptoms at age 66 years were improved by not vitamin B12 but vitamin B1, which suggested that the etiology of neuropathy was related to thiamine deficiency. Our patient had a medical history of chronic gastritis and gastric resection for cancer following hypothyroidism. Additionally, the patient was positive for serum anti-TG, anti-TPO, and anti-gastric parietal cell antibodies. Based on these findings, the patient was diagnosed with APS type 3B and neuropathy due to thiamine deficiency.

Vitamin B12 deficiency is a major symptom of APS type 3B [2,3]. Vitamin B12 can cause neurological symptoms including peripheral neuropathy and SCDS. In our case, at age 65 years, neuropathies were diagnosed based on neurological examination and neurophysiological tests. Additionally, the patient experienced vitamin B12 deficiency causing SCDS, and his symptoms were recovered by supplementation of vitamin B12. It seemed that the causes of severe vitamin B12 deficiency were gastric resection and anti-gastric parietal cell antibodies. SCDS due to vitamin B12 deficiency could cause numbness in the lower limbs, a feeling of stepping on cotton, walking instability, inability to stand smoothly after closing the eyes, paralysis, and limb tingling, which are caused by demyelination of the lateral and dorsal columns of the spinal cord [4]. When he visited our hospital for the second time, at age 66 years, we suspected that the gait disturbance was caused by a loss of position sense related to SCDS at first. However, this gait worsened despite vitamin B12 supplementation. Of note, after admission, although decreased serum vitamin B1 level was confirmed, supplementation with thiamine resulted in almost normal gait recovery. This fact suggested that the neurological symptoms were related to vitamin B1.

As far as we know, there is no previous report of APS type 3B with thiamine deficiency. One manifestation of APS type 3B is autoimmune thyroid disease. Here, the patient had a history of hypothyroidism related to anti-TG and anti-TPO antibodies and gastrectomy by gastric cancer. Classically, hypothyroidism has been reported to be associated with an increased susceptibility to gastric cancer [5]. Gastrectomy is a risk factor for thiamine deficiency, which could occur within four to six weeks postoperatively [6,7]. Moreover, thiamine transporters are highly expressed in the liver, stomach, and duodenum [8]. Therefore, gastrectomy may cause thiamine transporter dysfunction and lead to thiamine deficiency. Based on these findings, APS type 3B may be indirectly related to thiamine deficiency through the intercurrent diagnosis of hypothyroidism and gastric cancer. Additionally, he had a history of alcohol consumption. Alcohol consumption could decrease serum thiamine levels. Thiamine deficiency could cause Wernicke’s encephalitis and beriberi [9]. Severe thiamine deficiency may lead to axonal abnormalities and impaired acetylcholine transmission, which may result in ataxia, areflexia, and painful sensory or sensorimotor polyneuropathy, accompanied by severe degrees of muscle weakness [10]. Absent regular consumption, thiamine storage is depleted within two to three weeks, because thiamine has a short half-life ranging between one and 12 hours [11]. On the basis of these findings, we should pay attention to intercurrent diagnosis of thiamine deficiency in patients with APS type 3B, especially in cases with a history of alcohol consumption.

In our case, thiamine deficiency was suspected to have been caused by gastrectomy and alcohol consumption. In addition, we suspected that anorexia was a symptom of beriberi, which led to further reduction in food intake and thiamine deficiency. In fact, his food intake gradually decreased and he was unable to eat at all for a week before admission. Based on these findings, various manifestations, such as hypothyroidism; chronic gastritis; gastric cancer; vitamin B1 and B12 deficiency; and neuropathies may be directly and indirectly related to APS type 3B. 

## Conclusions

We encountered a rare case of APS type 3B with polyneuropathy due to thiamine deficiency. APS type 3B cases may be prone to subclinical vitamin B1 deficiency. Patients with APS type 3B may potentially become deficient in not only vitamin B12 but also vitamin B1 even if their dietary intake is good, and it is necessary to consider that thiamine deficiency could easily occur in cases of excessive alcohol consumption, eating difficulty or after gastrectomy.
